# CRISPRthripsis: The Risk of CRISPR/Cas9-induced Chromothripsis in Gene Therapy

**DOI:** 10.1093/stcltm/szac064

**Published:** 2022-09-01

**Authors:** Mario Amendola, Mégane Brusson, Annarita Miccio

**Affiliations:** Genethon, Evry, France; Integrare Research Unit UMR_S951, Université Paris-Saclay, Univ Evry, Inserm, Genethon, Evry, France; Laboratory of Chromatin and Gene Regulation during Development, INSERM UMR 1163, Université Paris Cité, Imagine Institute, Paris, France; Laboratory of Chromatin and Gene Regulation during Development, INSERM UMR 1163, Université Paris Cité, Imagine Institute, Paris, France

**Keywords:** genome editing, CRISPR/Cas9, chromothripsis, gene therapy, genotoxicity, micronuclei, chromosomal instability

## Abstract

The Clustered Regularly Interspaced Short Palindromic Repeats (CRISPR)/Cas9 nuclease system has allowed the generation of disease models and the development of therapeutic approaches for many genetic and non-genetic disorders. However, the generation of large genomic rearrangements has raised safety concerns for the clinical application of CRISPR/Cas9 nuclease approaches. Among these events, the formation of micronuclei and chromosome bridges due to chromosomal truncations can lead to massive genomic rearrangements localized to one or few chromosomes. This phenomenon, known as chromothripsis, was originally described in cancer cells, where it is believed to be caused by defective chromosome segregation during mitosis or DNA double-strand breaks. Here, we will discuss the factors influencing CRISPR/Cas9-induced chromothripsis, hereafter termed CRISPRthripsis, and its outcomes, the tools to characterize these events and strategies to minimize them.

## Graphical Abstract

**Figure:**
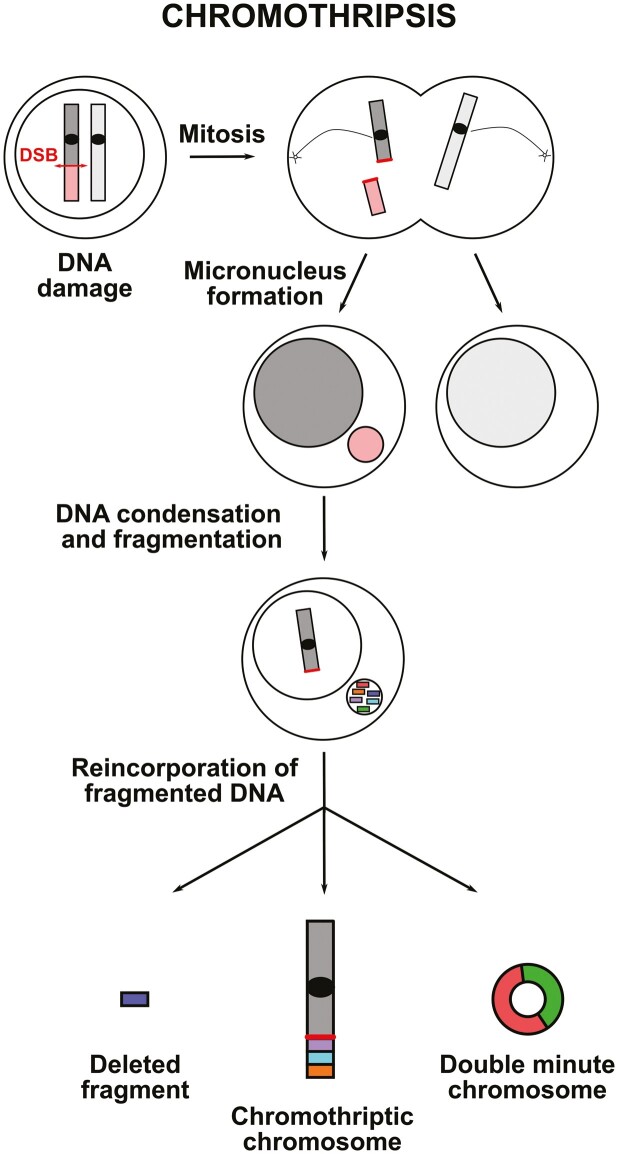
A DNA double-strand break can lead to the formation of acentric chromosome fragments and micronuclei. After DNA condensation, the chromosome fragment is shattered generating multiple DNA fragments, which are reincorporated in the nuclear genome forming a chromotriptic chromosome. If not reincorporated in the genome, these fragments can be lost (deleted fragments) or form double-minute chromosomes.

**Significance Statement**

The CRISPR/Cas9 system has revolutionized the field of gene therapy for genetic and non-genetic diseases allowing the generation of disease models and the development of effective treatments for numerous disorders. However, safety concerns on the use of CRISPR/Cas9 for clinical applications have emerged. Here, we will discuss recent findings on unanticipated catastrophic DNA rearrangements induced by CRISPR/Cas9 and their implications for gene therapy approaches.

## Introduction

The CRISPR/Cas9 system has revolutionized the field of gene therapy for genetic and non-genetic diseases allowing the generation of disease models and the development of effective treatments for numerous disorders. However, safety concerns on the use of CRISPR/Cas9 for clinical applications have emerged. Here, we will discuss recent findings on unanticipated catastrophic DNA rearrangements induced by CRISPR/Cas9 and their implications for gene therapy approaches.

## Gene Therapy Using Designer Nucleases

**Figure 1.**
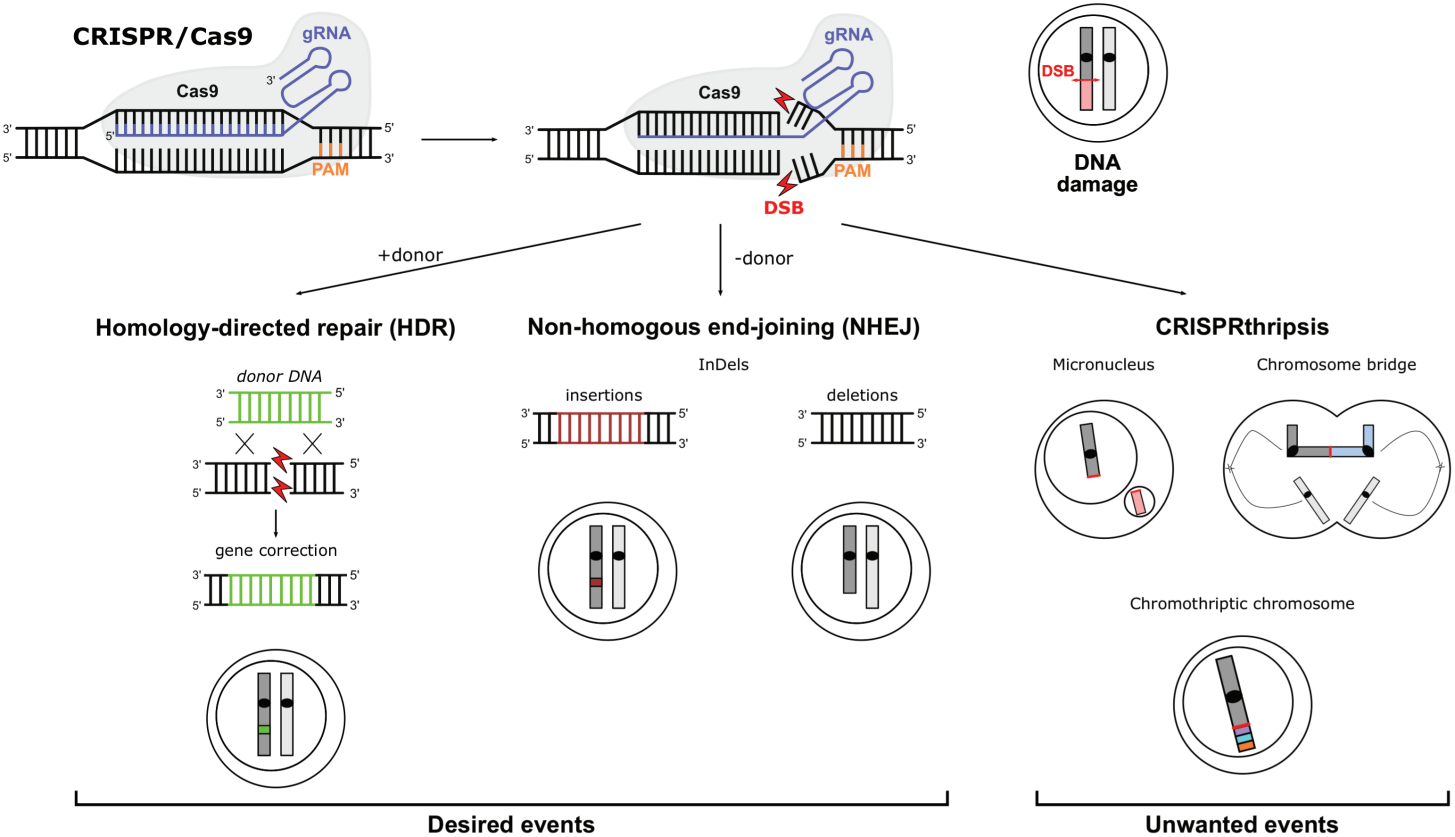
CRISPR/Cas9-induced events. The CRISPR/Cas9 complex is driven to a specific genomic site thanks to the complementarity of the gRNA to the target DNA region. Then, Cas9 induces a DNA double-strand break (DSB, red double arrow) 3-4 nucleotides upstream of the protospacer adjacent motif (PAM). This DSB can be repaired by the homology-directed repair (HDR) in the presence of a donor DNA or by non-homologous end joining (NHEJ) to generate InDels (insertion and deletions). However, if the DSB is not correctly repaired, one of the possible outcomes is CRISPRthripsis, with the formation of micronuclei containing an acentric chromosome fragment (light red box), chromosome bridges, and chromothriptic chromosomes (colored blocks represent shuffled DNA) (see Fig. 2).

Gene therapy was originally devised as a therapeutic replacement approach for monogenic disorders based on the delivery to the cells of a functional gene capable of compensating for the defective gene. Nowadays, the uses and indications are much broader, with most clinical trials concerning cancer treatment. There has been considerable diversification in the techniques, which are based on various corrective strategies, vectors, and methods including genome editing. Genome editing approaches use designer nucleases, such as the CRISPR/Cas9 nuclease system to induce DNA double-strand breaks (DSBs) via a guide RNA (gRNA) complementary to the genomic target (Fig. 1). The DSB can be repaired via homology-directed repair (HDR) by providing a donor DNA template containing the wild type sequence, allowing direct gene correction of the mutation. However, in the absence of integration of the DNA donor template, the DSB is simply repaired by the non-homologous end joining (NHEJ) pathway that usually generates short insertions or deletions (InDel). Other genome editing approaches leverage the NHEJ pathway to inactivate genes or *cis*-regulatory regions, eg, the enhancer of the *BCL11A* gene encoding a master transcriptional repressor of fetal hemoglobin (HbF) expression with the aim of reactivating HbF expression in adulthood and cure diseases characterized by deficient adult hemoglobin expression (namely beta-hemoglobinopathies).

Initial concerns on the use of the CRISPR/Cas9 system were focused on the potential off-target activity leading to unwanted generation of InDels in genomic regions other than the on-target site.^1^ Fortunately, numerous studies to understand the underpinning mechanism and strategies to predict, detect and reduce this risk have been proposed.^2^ Later, several groups have described that CRISPR/Cas9-induced DSB can be resolved in a complex and heterogeneous way, with the risk of inducing genomic rearrangements, such as large 1- to 50-kb deletions/inversions, translocations, chromosome loss, or chromosome truncations and combination of these rearrangements.^3, 4^ Overall, large deletions around the nuclease cutting site are the most common rearrangement and have been observed in mouse zygotes,^5, 6^ human embryos,^7, 8^ human and mouse embryonic stem cells^9^ and human hematopoietic stem cells (HSCs).^10^ Thanks to the use of long-read sequencing, in combination with long-range PCR and targeted sequencing,^11-13^ this outcome can be quantified and characterized at the nucleotide level. Very recently, CRISPR/Cas9-induced chromothripsis (hereafter termed CRISPRthripsis; Fig. 1) has been described in cell lines.^14^ Unfortunately, unlike off-targets, these on-target risks cannot be reduced by more specific DSB approaches.

## Chromoanagenesis or Genomic Chaos

**Figure 2.**
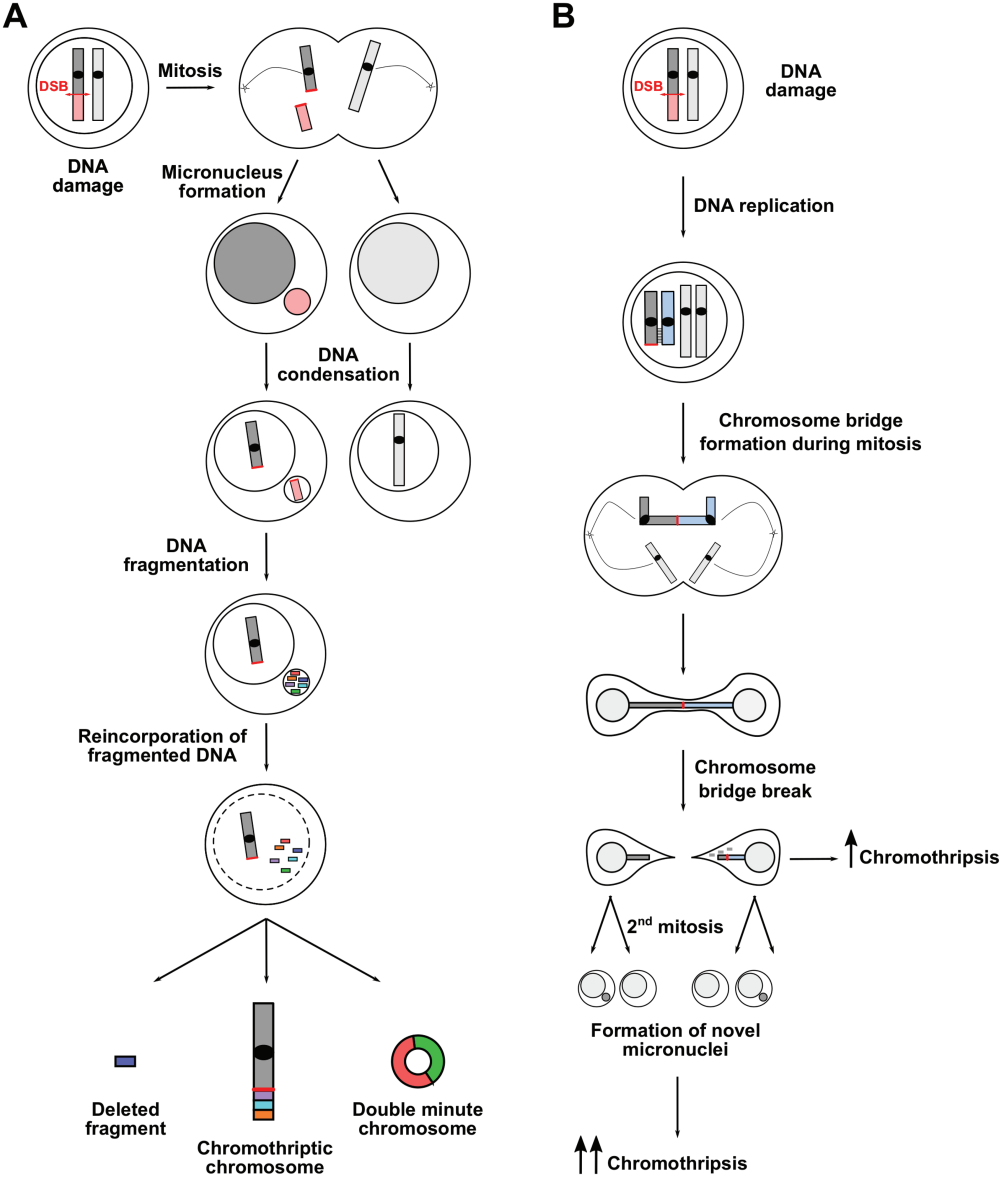
Chromothripsis leads to massive genomic rearrangements. (A) A DNA double-strand break (DSB, red double arrow) can lead to the formation of an acentric chromosome fragment (light red box). After mitosis, this fragment can be enwrapped by a lipid membrane forming a micronucleus (red circle). After DNA condensation, the chromosome fragment is shattered generating multiple DNA fragments, which are eventually reassembled and reincorporated into the nuclear genome forming a chromotriptic chromosome. If not reincorporated in the genome, these fragments can be lost (deleted fragments) or form double-minute chromosomes. (B) A DNA DSB can lead to the formation of sister chromatids with shortened or absent telomeres that form a chromosomal bridge. During the first mitosis, cell division leads to breakage of the chromosomal bridge, which can induce local DNA fragmentation and chromothripsis. During the second mitosis, the broken chromosome missegregates, potentially leading to the formation of micronuclei, which also trigger chromothripsis (see panel A).

Chromothripsis was firstly described in cancer cells as a process characterized by the occurrence of massive chromosomal rearrangements usually clustered on one or few chromosomes.^15^ In fact, although these events can lead to cell death, they can also have a role in malignant transformation. Chromothripsis is observed with a frequency of >50% in several cancers^16^ and is emerging as a predictor of negative clinical outcome. The mechanisms underlying chromothripsis are not fully elucidated. It is believed that DNA fragmentation is followed by the formation of micronuclei containing an entire chromosome or part of a chromosome that are further fragmented, reassembled, and eventually incorporated into the genome of the nucleus in the following mitoses^17^ (Fig. 2A). Chromothripsis can also occur as a consequence of the formation of chromosomal bridges during mitosis due to the fusion of the two sister chromatids with telomeric loss^18^ (Fig. 2B). Finally, chromothripsis can generate double minute chromosomes, small circular acentric chromosomes that can be present at very high copy number and carry oncogenes, thus promoting tumor development.^19^

Nowadays, it is clear that the chromothripsis mechanism could not account for all the phenomena of rapid chromosomal rearrangements arising during single chaotic cellular events. In fact, these catastrophic phenomena include not only chromothripsis, but also chromoanasynthesis and chromoplexis, and are grouped under the name of “chromoanagenesis” (chromosome regeneration/evolution).^20^ Chromoanasynthesis (chromosome resynthesis) results in localized complex rearrangements with duplication and triplication of a single chromosome due to erroneous DNA replication,^21^ and chromoplexy (chromosome restructuring) refers to the occurrence of multiple inter- and intra-chromosomal translocations and deletions with little or no copy number alterations.^22^

## Safety Concerns of Nuclease-based Editing Approaches: Micronuclei, Chromosomal Bridges, and CRISPRthripsis

Recently, CRISPR/Cas9-induced chromothripsis (CRISPRthripsis) has been described in cell lines^14^ (Fig. 1). In addition, the formation of micronuclei and chromosomal bridges due to the on-target cleavage has also been observed in primary cells including mouse embryos and human hematopoietic stem/progenitor cells (HSPCs), the target cell population in gene therapy approaches for many hematopoietic and non-hematopoietic disorders^14, 23^ (Fig. 1). In particular, the formation of micronuclei was observed in 20%-30% of mouse embryos and 2.5% of human HSPCs edited with Cas9 targeting the *BCL11A* enhancer, a proposed clinical target for beta-hemoglobinopathies^14, 24^ (NCT03655678 and NCT03745287). The analysis of micronuclei in the drug product used in these clinical trials^15^ was not reported. However, CRISPRthripsis was observed neither in mouse embryos nor in human HSPCs, probably because only a small fraction of cells containing micronuclei undergo CRISPRthripsis and the number of cells analyzed in these studies was likely too low to observe this event. Furthermore, some cells can undergo cell death after CRISPRthripsis. However, even a rare event leading rather to a malignant transformation could be a concern in many gene therapy approaches where millions of cells are targeted. Fortunately, to date, no genotoxic events were reported in the NCT03655678 and NCT03745287 clinical trials.

## Tools to Study Micronuclei and Chromosomal Bridges Formation and CRISPRthripsis

Micronuclei are defined as small-sized nuclei between 1/20th and 1/5th of the size of the main nucleus^25^ containing one/few chromosomes or chromosome fragments.^26, 27^ Micronuclei differ from nuclei in terms of chromatin condensation, nuclear envelope composition, and the absence of proteasomes.^28^ Since micronuclei are considered a reliable readout of genomic instability, several approaches to quantify and characterize them have been described. *In primis*, nuclear and chromosomal alterations, such as micronuclei and chromothripsis, can be studied with classical and modern cytogenetics approaches, like GTG banding^29^ and fluorescence in situ hybridization,^30^ which enables a whole genomic view in a cost-efficient and single-cell oriented fashion. A special case of fluorescence hybridization is comparative genomic hybridization (CGH array), which allows genome copy number variation analysis.^31^ However, the resolution of these methods is limited to kilo- to mega-base pair. An exception is molecular combing, which consists in performing FISH after combing high molecular weight DNA on a glass surface.^32^ An overview of chromosomes’ spatial organization and interactions can be obtained via high-throughput chromosome conformation capture approaches, which can provide both a multi-cell-based genomic view or at a single-cell level.^33-35^

Numbers, shape, and size of micronuclei, and chromosome bridges can be studied via imaging using either microscopy^36, 37^ or imaging flow cytometry.^38, 39^ Beyond frequency, size of micronuclei represents an important feature since there is a strong correlation between the size of the micronucleus and the presence of centromere, with centromere-positive micronuclei being larger than centromere-negative micronuclei.^40^ Recent development of flow imaging will allow enrichment of rare micronuclei-containing cells for subsequent analysis.^41^ In addition, novel techniques allowing purification of micronuclei and sequencing, at both population and single-cell levels, will provide a systematic approach to study genomic instability and reveal novel molecular details in the process.^42^

Finally, “Look-Seq” is a powerful technique for tracking cells containing micronuclei and subjecting their progeny to single-cell sequencing in order to understand the evolution and fate of micronuclei and cells bearing them.^14^

## Factors Influencing Micronuclei and Chromosomal Bridges Formation and CRISPRthripsis and Their Outcomes

There are several factors that can influence the formation of micronuclei and chromosome bridges, and CRISPRthripsis and determine their consequences.

First, these events occur in dividing cells, therefore the genotoxic risk is theoretically minimized or abolished in quiescent and non-dividing cells. By way of example, HSPCs are a mixed population of short-term progenitors and HSCs, which will sustain in the long-term the hematopoietic system in the treated patients. HSCs are mostly quiescent and therefore the risk of such chromosomal rearrangements is low. HDR-based approaches for gene correction require a long stimulation of the cell cycle (72-96 hours) as HDR occurs only in dividing cells. Therefore, when possible, NHEJ-based approaches should be preferred as NHEJ occurs in all the phases of the cell cycle. Nevertheless, it is difficult to assess the cell cycle in *bona fide* HSCs at the time of transplantation as they are difficult to identify, and we cannot exclude that even a short-term treatment (typically 48 hours for NHEJ-based approaches) can induce the cell cycle in HSCs. Finally, HSPC-based gene therapy involves the transplantation of proliferating hematopoietic progenitors together with HSCs. If such chromosomal rearrangements occur in progenitor cells, we cannot exclude the development of malignant clones from this cell population.

Furthermore, an important consideration is that the occurrence and frequency of these rearrangements can vary across tissues and cell types according to their response to DNA DSBs,^43^ eg, between HSCs and hematopoietic progenitors.^44^

The probability of these events is also specific to each CRISPR/Cas9-based therapeutic strategy and the number of DSB that are generated per cell, eg, editing of multiple genes (gamma-^45^ or alpha-globin^46^ genes) or performing genomic deletions (beta-^47^ or alpha-globin^48^ genes) will introduce more than one DSB per chromosome.

Moreover, the chromatin context of the target site can also influence the choice of DNA repair pathway and thus the occurrence of these massive genomic rearrangements. By way of example, locus-specific differences in the chromatin status or in DNA repair efficiency^49^ can determine if DSBs are correctly repaired or if they lead to loss of part of a chromosome, and as a consequence formation of micronuclei, chromosome bridges, and CRISPRthripsis. Similarly, the chromosomal location of the target site can also affect the outcome of these events in terms of cell death or clonal expansion, depending on the number and nature of genes present in the target chromosomes (eg, oncogenes and tumor suppressor genes).

Finally, another important factor is the presence of TP53. In fact, while TP53 does not influence the formation of micronuclei, it inhibits the division of around 50% of micronucleated cells, thus potentially avoiding the occurrence of CRISPRthripsis in the following mitosis.^14, 50^ Therefore, transient TP53 inhibition proposed to increase HDR efficiency by minimizing apoptosis of edited cells^51^ should be considered in view of a possible increase in the occurrence of the CRISPRthripsis.^52^

Overall, the occurrence of these catastrophic events should be closely monitored in pre-clinical gene therapy studies. The recent studies described the occurrence of these events in an HSC-based gene therapy product potentially used for many different diseases.^52^ However, long-term in vitro and in vivo experiments in the specific target cell types using highly sensitive tools are required to assess the safety of CRISPR/Cas9 nuclease-based gene therapy approaches.

## Strategies to Minimize Micronuclei and Chromosomal Bridges Formation and CRISPRthripsis

To reduce the possibility of such complex genomic rearrangements, gene therapy approaches should: (i) reduce the number of edited cells by stringent selection of target cells (eg, only real HSCs^53^); (ii) reduce cell cycling or restrict editing to the G1 phase of the cell cycle^54-56^; (iii) reduce additional stress associated with cell manipulation^57, 58^; (iv) target the “right” genomic harbor for each application, eg, to achieve high transgene expression with a limited number of edited cells^59^ or to avoid chromosomal regions enriched in oncogenes or oncosuppressors; (v) reduce large genomic rearrangements due to repeated DNA cleavage of seamless repaired DSB, by modulating DNA repair^60^ or by reducing exposure time to nucleases using protein delivery.^61^

In addition, approaches based on the use of dead Cas9 or Cas9 nickase (eg, base editing, prime editing, and epigenome editing) minimize the formation of DSB and, likely, the generation of chromosomal bridges and the occurrence of CRISPRthripsis. However, there is still a risk of DSB when using technologies using Cas9 nickase (eg, base and prime editing), therefore the occurrence of these rearrangements should be investigated when there is evidence of DSB occurring after Cas9 nickase treatment.

## Summary/Conclusion

Chromotripsis is a major driver of extrachromosomal DNA,^62^ thus representing an important safety issue for gene and cell therapy that needs to be carefully addressed. Although occurring at low frequency, it is a dangerous event considering the large number of cells that need to be modified to achieve a clinical benefit. In addition, chromothripsis is not the only genome catastrophe that can happen. According to in vitro models, chromothripsis makes up roughly <10% of all different types of chaotic genomes identified.^63, 64^ Besides the occurrence of chromoanasynthesis and chromoplexy, various types of cell death, including mitotic cell death,^65-67^ apoptosis,^68, 69^ necroptosis—a programmed version of necrosis^70^ and entosis^71^ can all reverse their own process, causing genomic alterations in surviving cells. The newly available technologies and the sudden interest brought to the domain by the CRISPRthripsis phenomenon promise to elucidate in the near future the mechanism causing chromothripsis, micronuclei, and genomic chaos, and their consequences on the surviving cell population, the end product that really matters for gene therapy.

## Data Availability

No new data were generated or analyzed in support of this research.
